# Giant electro-optic effect in Ge/SiGe coupled quantum wells

**DOI:** 10.1038/srep15398

**Published:** 2015-10-19

**Authors:** Jacopo Frigerio, Vladyslav Vakarin, Papichaya Chaisakul, Marcello Ferretto, Daniel Chrastina, Xavier Le Roux, Laurent Vivien, Giovanni Isella, Delphine Marris-Morini

**Affiliations:** 1 L-NESS, Dipartimento di Fisica, Politecnico di Milano, Polo di Como, Via Anzani 42, I 22100 Como, Italy; 2 Institut d’Electronique Fondamentale, Univ. Paris-Sud, CNRS UMR 8622, Bât. 220, 91405 Orsay Cedex, France

## Abstract

Silicon-based photonics is now considered as the photonic platform for the next generation of on-chip communications. However, the development of compact and low power consumption optical modulators is still challenging. Here we report a giant electro-optic effect in Ge/SiGe coupled quantum wells. This promising effect is based on an anomalous quantum-confined Stark effect due to the separate confinement of electrons and holes in the Ge/SiGe coupled quantum wells. This phenomenon can be exploited to strongly enhance optical modulator performance with respect to the standard approaches developed so far in silicon photonics. We have measured a refractive index variation up to 2.3 × 10^−3^ under a bias voltage of 1.5 V, with an associated modulation efficiency V_π_L_π_ of 0.046 V cm. This demonstration paves the way for the development of efficient and high-speed phase modulators based on the Ge/SiGe material system.

Silicon photonics has generated strong advances in recent years for on-chip optical communications. Silicon based-optoelectronic devices have been intensively studied and the recent advances proved the capability of silicon photonics to offer some viable solutions for many applications including optical telecommunications and optical interconnects. In this context Ge rich-Ge/SiGe quantum wells (QW) have received a growing interest since the first demonstration of the quantum-confined Stark effect (QCSE) in these structures in 2005^1^. The QCSE causes a red-shift of the absorption spectrum and a significant reduction of the excitonic absorption peak^2^; features that can be exploited for optical intensity modulation. This result paved the way to a number of exciting works addressing the absorption mechanisms in Ge/SiGe QW structures and tackling the fabrication of innovative optoelectronic devices in the near-IR wavelength range (1.3 μm-1.5 μm). Compact and efficient intensity modulators at wavelengths of 1490 nm^3^,^4^,^5^,^6^ and 1550 nm^7^,^8^ have been demonstrated as well as high-speed behaviour^9^. The most striking feature of electro-absorption modulators based on the QCSE is the possibility to reach power consumption lower than 100 fJ/bit, as demonstrated in^9^, thus meeting the aggressive requirements for on-chip optical interconnects^10^. As a major advantage, the compositions and thicknesses of the layers in a Ge/SiGe QW can be engineered to modify the electro-optical properties, such as to address modulation at 1.3 μm wavelength^11^,^12^,^13^. All the aforementioned modulators works by exploiting electroabsorption based on the QCSE of excitonic transitions between the first valence band heavy hole or light hole level (HH1 or LH1), and the first conduction state at Γ (cΓ1). In contrast, much less research effort has been spent on investigating the change in Ge/SiGe QW refractive index due to the variations of their absorption spectra, i.e. electrorefraction, as given by the Kramers-Kronig relations. Indeed only recently, an experimental value of effective refractive index variation of 1.3 × 10^−3^ under −8 V, with a figure merit V_π_L_π_ of 0.46 Vcm was reported for Ge/Si_0.15_Ge_0.85_ QW^14^. In this preliminary demonstration, the voltage required to induce electro-refraction effect was too high to be compatible with the voltage delivered by CMOS microelectronic circuits. However, the electro-optical properties of quantum well structures can be further tailored by coupling quantum wells together. Coupled quantum wells (CQW) exhibit interesting physical behaviours due to the coupling of the wave-functions through the barrier, allowing an ultimate control of the electro-optical properties. For this reason, in III-V semiconductors, symmetric and asymmetric CQW have been widely investigated for many purposes including non-linear optics^15^, infrared photodetection^16^, THz emission^17^ and low-voltage optical modulation^18^. Theoretically, symmetrically Ge/SiGe CQW have been proposed to enhance the electro-refractive effect^19^. A surfaced-illuminated Ge CQW device was recently fabricated^20^; in this case the electro-absorption was studied through resistivity measurements. In this work we report the first experimental demonstration of Ge/SiGe CQW waveguide showing an anomalous QCSE and a strong electro-refraction effect under low bias voltage. The electro-optical properties of Ge/SiGe CQWs have been experimentally investigated by means of optical transmission measurements of a Fabry Perot cavity. The absorption spectra analysis and the comparison with theoretical modelling shows the influence of the coupling of the quantum wells and the necessity to consider the two first electron and heavy hole confined states to understand the experimental features. Electro-refraction is deduced from the Fabry Perot resonance shifts as a function of the applied electrical field. The results have been used to evaluate the characteristics of a phase-shifter based on this structure.

## Results

### Device design

**Figure 1:**
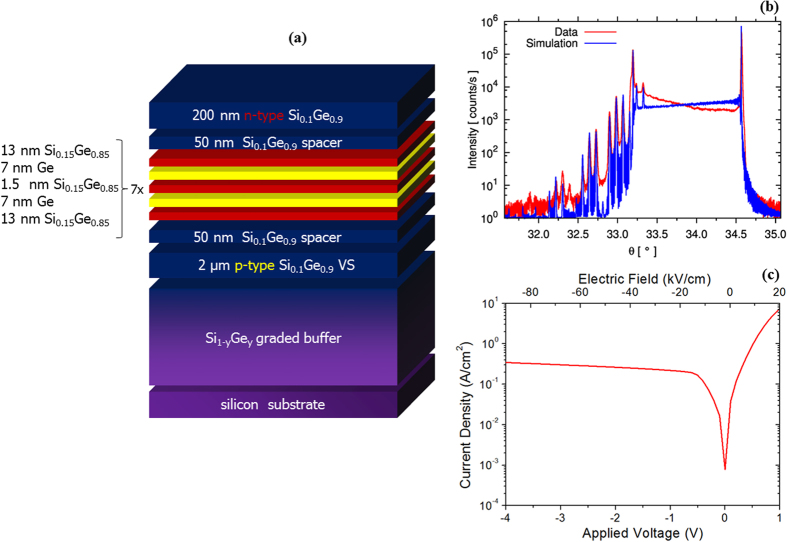
(**a**) Cross section of the sample showing the Ge/SiGe CQW between n-doped SiGe and p-doped SiGe to form a PIN diode. The stack is grown on a Si substrate via a graded buffer by LEPECVD. (**b**) X-ray diffraction: the agreement between experimental and simulated ω-2θ scans around the(004) reflection confirms that the CQW structure has been realized. (**c**) Current density of the device as a function of the applied voltage.

The CQW heterostructure was grown by low-energy plasma-enhanced chemical vapour deposition (LEPECVD)^21^. A cross section of the structure is shown in Fig. 1(a). The CQW consists of seven pairs of CQWs: 7 × [7 nm Ge QW + 1.5 nm Si_0.15_Ge_0.85_ inner barrier + 7 nm Ge QW + 26 nm Si_0.15_Ge_0.85_ outer barrier]. Individual layer thicknesses and compositions were designed to obtain a strain-symmetrized structure. The outer barrier is thick enough to avoid coupling between adjacent periods. The symmetric ω-2θ scan (taken through the Si(004) Bragg peak) is reported in Fig. 1(b). The good agreement between the experimental and the dynamically simulated X-ray diffraction spectra confirms the presence of the thin Si_0.15_Ge_0.85_ inner barrier. The CQW are embedded in a PIN diode in order to apply an electrical field perpendicular to the CQW planes. In Fig. 1(c) is reported the current density as a function of the applied voltage and of the electric field.

### Electro-Refraction measurement in CQW

**Figure 2:**
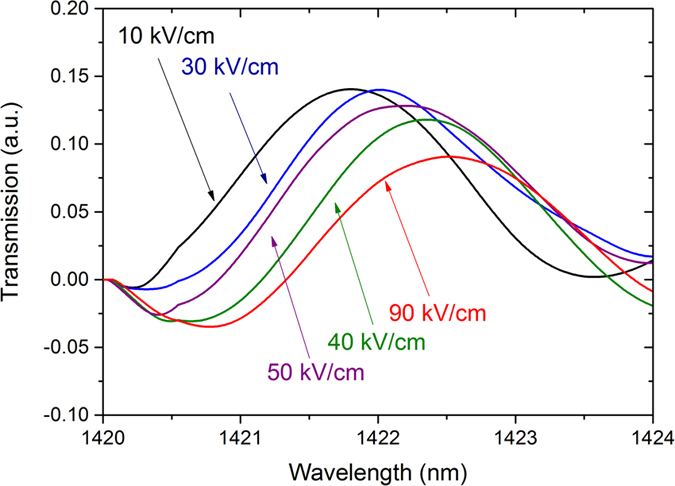
Electro-refraction measurement. The wavelength dependence of the transmission is characteristic to the Fabry Perot effect induced by multiple reflections at the waveguide facets.The shift of the fringes with the applied electric field is related to the refractive index variation in the CQW structure.

**Figure 3:**
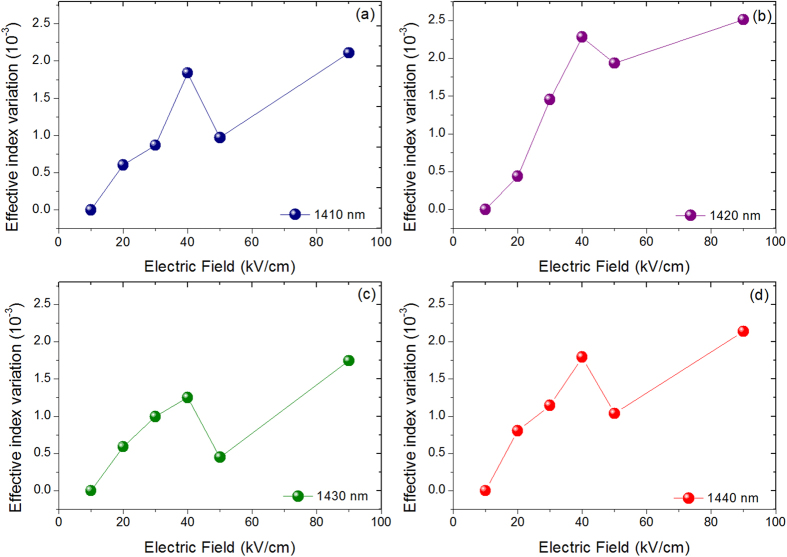
Effective index variation as a function of the electric field at 1410 nm (**a**), 1420 nm (**b**), 1430 nm (**c**) and 1440 nm (**d**).

**Figure 4:**
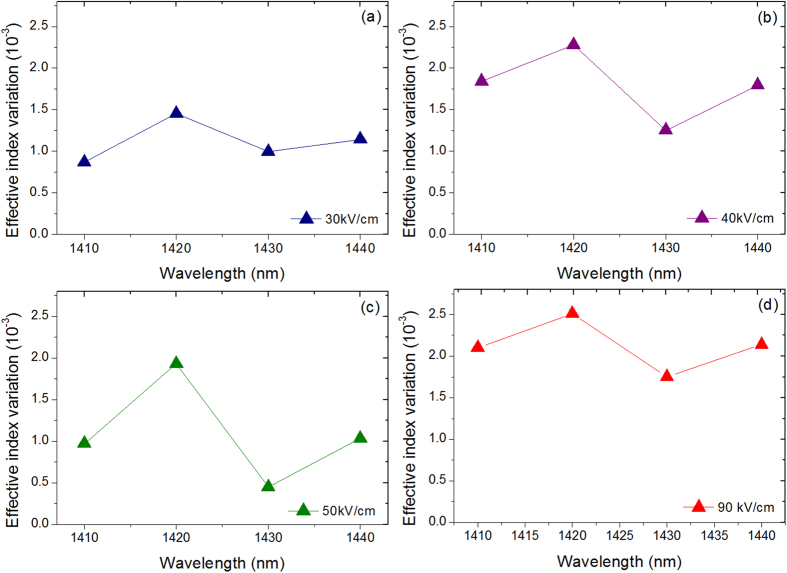
Effective index variation as a function of the wavelength at 30 kV/cm (**a**), 40 kV/cm (**b**), 50 kV/cm (**c**) and 90 kV/cm (**d**).

64 μm long planar waveguide has been fabricated to investigate the electrorefraction in Ge/SiGe CQW. At wavelengths larger than the absorption band-edge (located around 1400 nm), Fabry Perot fringes induced by multiple reflections at the waveguide facets have been analyzed. As an example, a Fabry Perot fringe around 1422 nm and its shift as a function of the electrical field is reported in Fig. 2. The effective index variation was experimentally deduced at different wavelengths, using the method described in ref. 14. The measured effective index variation as a function of the electric field is reported in Fig. 3 for different wavelengths above the absorption band-edge. Due to the built-in potential, an electric field of 10 kV/cm is present when the applied bias voltage is 0 V. Strikingly, the refractive index variation shows a local maximum at an electric field of 40 kV/cm for all considered wavelengths. Conversely, in a standard non-coupled quantum well the effective index increased quadratically with the electrical field^14^. Interestingly, the effective index variation also shows a maximum at a wavelength of 1420 nm for all the considered electric fields (see Fig. 4), while in standard QW it continuously decrease as the wavelength increases^14^. As a consequence, CQW structures can be specially tailored for optimized operation at low-bias for different spectral region. In the following section, the absorption spectra of Ge/SiGe CQW have been measured and theoretically analyzed to explain the anomalous behavior of the effective index variation with the applied electric field and as a function of the wavelength.

### Absorption spectra analysis

**Figure 5:**
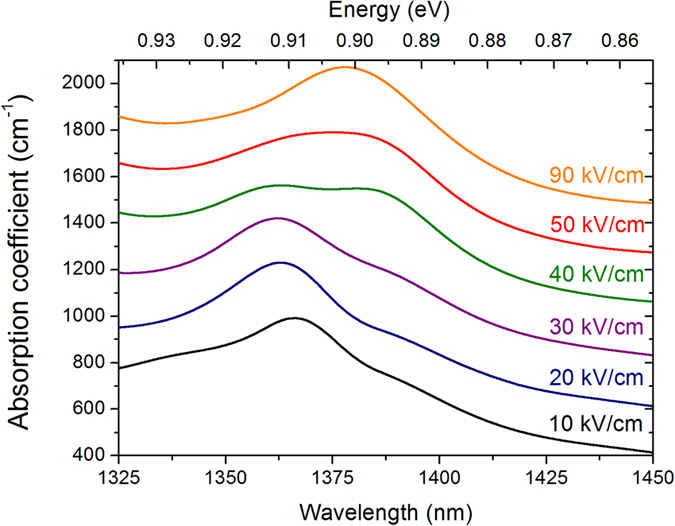
Absorption spectra as a function of the applied electric field. The data have been filtered and the baseline of each curve has been shifted of 200 cm^−1^.

**Figure 6:**
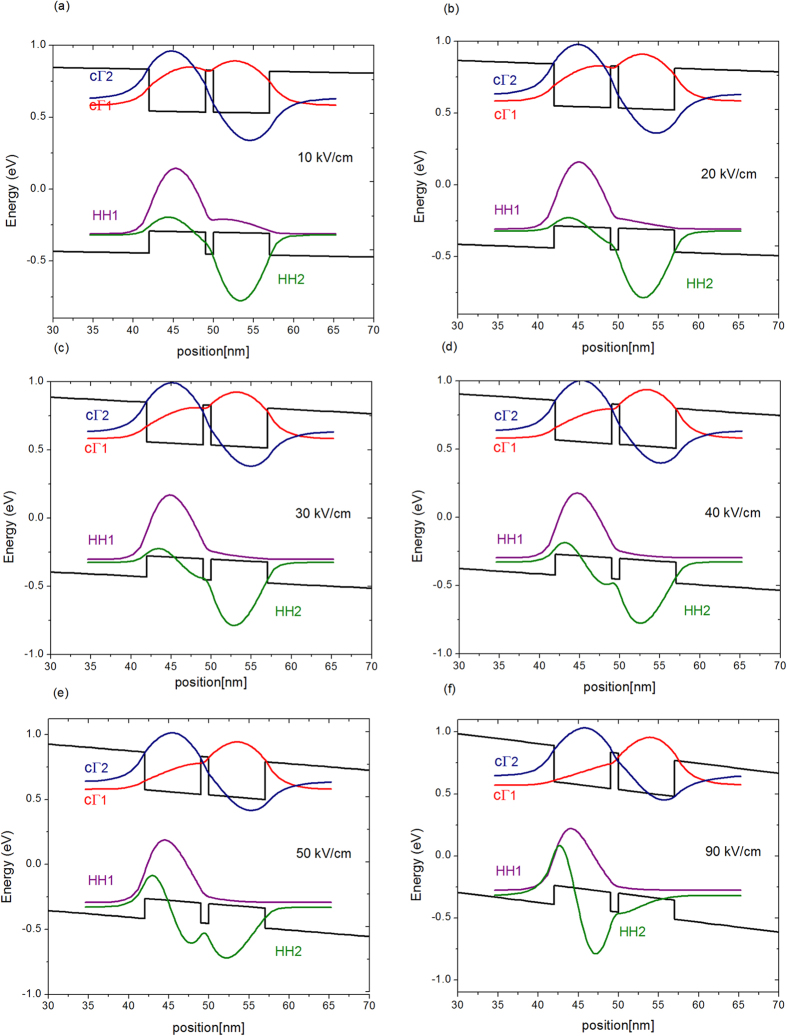
Simulated wavefunctions in the CQW at Γ point, of the cΓ1, cΓ2, HH1 and HH2 with an electric field of: (**a**) 10 kV/cm, (**b**) 20 kV/cm, (**c**) 30 kV/cm, (**d**) 40 kV/cm, (**e**) 50 kV/cm and (**f**) 90 kV/cm.

The absorption spectra of the device at different reverse bias voltages obtained from optical transmission measurements are reported in Fig. 5 for TE polarization. The data have been filtered and the baseline of each curve has been shifted by 200 cm^−1^ to better highlight the spectral features. From these measurements the absorption spectra in Ge/SiGe CQW show unique characteristics which differ from those previously recorded in standard non-coupled Ge/SiGe QW^14^. A combination of increasing/decreasing absorption peaks is obtained when the electrical field varies from 10 kV/cm to 90 kV/cm. To understand this behavior, the measurements are compared with simulations performed within the envelope function approximation using the Nextnano software package^22^. The simulated wavefunctions of cΓ_1_, cΓ_2_, HH_1_ and HH_2_ in the CQW as a function of the applied electric field are reported in Fig. 6. In a CQW, the cΓ_1_ (resp. HH_1_) and cΓ_2_ (resp. HH_2_) transitions represents the “bonding” (zero-node wavefunction) and “anti-bonding” (one-node wavefunction) which are formed due to the QW coupling. In such a system, the electric field breaks the symmetry, thus the optical transitions HH_2_-cΓ_1_ and HH_1_-cΓ_2_ are no longer forbidden by selection rules as in standard uncoupled quantum wells and consequently the absorption spectra, in the considered energy range, consist of a combination of four optical transition: HH_1_-cΓ_1_ (T_11_), HH_2_-cΓ_2_ (T_22_), HH_2_-cΓ_1_ (T_12_) and HH_1_-cΓ_2_ (T_21_). At 10 kV/cm the cΓ_1_ and cΓ_2_ wavefunctions are still delocalized on both wells, while the HH_1_ and HH_2_ wavefunctions show a significative localization in the left and right wells respectively (see Fig. 6a). The absorption spectrum at 10 kV/cm is dominated by two spectral features: a peak located at 0.909 eV and a shoulder at lower energy which are formed by a superposition between the T_22_ and T_21_ transitions and between the T_11_ and T_12_ transitions respectively. The simulations show that, as the field increases, the spatial overlap between the wavefunctions (and consequently the intensity of the associated optical transition) of T_12_ and T_21_ increases, while that of T_11_ and T_22_ decreases. Moreover, as the electric field increases, the T_22_ and T_12_ transitions red-shift while the T_11_ and T_21_ transitions are blue-shifting. As a consequence, a clear blue-shift of the high energy peak is observed in the absorption spectra recorded at 20 kV/cm and at 30 kV/cm. The intensity of the low energy spectral feature increases because there is an increasing spectral overlap between the T_12_ and T_21_ transitions. At 40 kV/cm the absorption spectrum is dominated by two peaks clearly recognizable at 0.912 eV and at 0.897 eV. The simulations show that the spatial overlap between the wavefunctions associated to the T_11_ and T_22_ become vanishingly small at 40 kV/cm, and consequently the two peaks have been attributed to the T_21_ and T_12_ transitions. As the electric field increases, the spectral overlap between the T_21_ and T_12_ transitions increases, and at 90 kV/cm, only a single broad peak is visible in the absorption spectrum. As a conclusion of this analysis, the unusual behavior of the QCSE in CQW is explained by the evolution of both electron and hole wavefunctions in the CQW as a function of the electric field. The number of excitonic transition and the strong evolution of the excitonic peak intensities near the absorption band-edge are quite different from the normal QCSE, which explains the large electrorefraction and its non-usual wavelength dependence measured in this structure.

## Discussion

The anomalous CQSE in the Ge/SiGe CQW structure leads to effective index variations of more than 2 × 10^−3^ under an electrical field of only 40 kV/cm. In comparison, in classical uncoupled Ge/SiGe QW structures an effective index variation of 1.3 × 10^−3^ was measured with an applied electric field of 88 kV/cm. Using the CQW structure, under a bias voltage as low as 1.5 V (corresponding to an electric field of 40 kV/cm) on a 300 μm-long structure is enough to obtain a π phase shift. A V_π_L_π_ figure of merit of only 0.046 V cm can then be deduced, which is one order of magnitude better than in uncoupled Ge/SiGe QWs^14^ and very competitive with respect to Si-based phase shifters^23^,^24^. In addition the V_π_L_π_ figure of merit can be further improved by increasing the overlap between the optical mode and the QW region (which was 17% in this work). Optimizing this overlap will also allow a reduction of the optical losses caused by the thick p-doped SiGe virtual substrate in this tested structure. To fully exploit the potentiality of Ge/SiGe CQW as compact, high speed and low power consumption optical modulators, the active region will have to be integrated into an interferometric structure such as a Mach– Zehnder interferometer. It can be noticed that the integration of QW active regions with low-loss SiGe waveguides on top of graded buffer is a promising approach, as the integration of a passive SiGe waveguide with an electro-absorption modulator and a modulator was demonstrated recently^25^. Remarkably, all the devices were monolithically integrated on silicon by a single epitaxial growth. Finally besides the interest for low power consumption optical modulators, the control of quantum confinement in Ge/SiGe CQW paves the way of a new route towards the exploitation of nonlinear optical effects in Ge/SiGe.

## Methods

### Epitaxial growth and characterization

The CQW heterostructure was grown by low-energy plasma-enhanced chemical vapor deposition (LEPECVD)^21^ on a 100 mm n-Si(001) substrate with a resistivity of 1–10 Ω cm. Before the heteroepitaxial growth, the substrate was dipped in an aqueous hydrofluoric acid solution for 30 seconds to remove the native oxide. The first part of the structure consists of a Si_1-y_Ge_y_ graded buffer, with a total thickness of 13 μm, where the Ge concentration y was linearly raised from 0% to 90% with a grading rate of 7%/μm. The growth rate was 5–10 nm/s, while the substrate temperature was linearly decreased from 740 °C to 525 °C . The graded buffer was then capped with a 2 μm thick p-doped (5 × 10^18^ cm^−3^) Si_0.1_Ge_0.9_ layer to form a fully relaxed virtual substrate (VS) and the p-type contact of the p-i-n structure embedding the CQWs. The threading dislocation density was 6 × 10^6^ cm^−2^ as measured by chemical defect etching^26^. The CQW consists of seven repetitions of the following period: 7 nm Ge well +1.5 nm Si_0.15_Ge_0.85_ inner barrier +7 nm Ge well +26 nm Si_0.15_Ge_0.85_ outer barrier. The CQW structure was grown at rate of 1 nm/s and the temperature was lowered at 475 °C to avoid the interdiffusion at the barrier/QW interfaces. Finally, a 200 nm phosphorous doped (1 × 10^19^ cm^−3^) Si_0.1_Ge_0.9_ n-type contact layer was deposited. Layer compositions and strain states were measured by high-resolution x-ray diffraction (HR-XRD) using a PANalytical X’Pert PRO MRD diffractometer. Out-of-plane and in-plane lattice parameters were measured (relative to the Si (004) reflection) for the VS peak and the superlattice satellites. Ge content and strain were then obtained using the known lattice parameters for relaxed SiGe alloys^27^ and interpolated elastic constants of Si and Ge^28^. The final composition of the VS was found to be 90% (with a residual in-plane strain of 0.12%). The in-plane lattice parameter of the CQW stack is the same as that of the VS, meaning that the CQW stack is coherently matched to the VS.

### Device fabrication

The waveguides were patterned by dry etching to the p-doped Si_0.1_Ge_0.9_ layer. The sidewall roughness of the etched mesa was smoothened by hydrogen peroxide (H_2_O_2_) solution. 100 nm of silicon dioxide were deposited as passivation layer on the left and right walls of the waveguide by plasma-enhanced chemical vapor deposition (PECVD). For n and p contacts 10 nm of Ti and 300 nm of Au was evaporated and lifted off.

### Optical transmission measurements

The measurements were performed at room temperature with spectral resolution of 0.1 nm. A tunable laser from 1325 to 1450 nm with an output power of 1 mW was used. Light from the laser was butt coupled into the waveguide using a lensed fiber. Light was injected into the waveguide part which was not covered by the metal to minimize optical losses. An objective was used to couple the output light into a photodetector. Light polarization was controlled at the input and the output of the waveguide. The transmission of the device is normalized by the transmission of the set-up to take into account its wavelength dependence. Calculations have been performed in order to investigate the effect of the air/waveguide reflection on the transmission spectra. A homogenized medium of Si_0.1_Ge_0.9_ is considered on top of the graded buffer, with a refractive index of 4.228 at 1.32 μm and 4.209 at 1.45 μm. Effective indexes of 4.222 and 4.202 are obtained at the wavelengths of 1.32 μm and 1.45 μm, respectively. A reflection of 38.07% is obtained at 1.32 μm and 37.89% at 1.45 μm. The corresponding losses difference is thus around 0.02 dB, thus the effect of the air/waveguide reflection on the the transmission spectra is negligible.

## Additional Information

**How to cite this article**: Frigerio, J. *et al.* Giant electro-optic effect in Ge/SiGe coupled quantum wells. *Sci. Rep.* **5**, 15398; doi: 10.1038/srep15398 (2015).
